# Effects of High-Intensity Interval Training (HIIT) on Physical Performance in Female Team Sports: A Systematic Review

**DOI:** 10.1186/s40798-023-00623-2

**Published:** 2023-08-18

**Authors:** Mima Stankovic, Dusan Djordjevic, Nebojsa Trajkovic, Zoran Milanovic

**Affiliations:** 1 Faculty of Sport and Physical Education, University of Niš, Čarnojevičeva 10a, 18000 Niš, Serbia; 2 Science and Research Centre, Institute for Kinesiology Research, Garibaldijeva 1, 6000 Koper, Slovenia; 3 Faculty of Sports Studies, Incubator of Kinanthropological Research, Masaryk University, 60200 Brno, Czech Republic

**Keywords:** Interval training, Output, VO_2_max, Physical fitness

## Abstract

**Background:**

There is limited information regarding adaptation of HIIT in female athletes which is important since the adaptation to HIIT may be different compared to male athletes. Therefore, the aim of this systematic review was to summarize the effects of HIIT on physical performance in female team sports athletes.

**Methods:**

The following databases Google Scholar, PubMed, Web of Science, Cochrane Library, ProQuest and Science Direct were searched prior to September 2nd, 2022. The inclusion criteria were longitudinal studies written in English, elite, sub-elite or college female team sports participants, and HIIT intensity had to be at 80–100% maximal heart rate. There were no exclusion criteria regarding the age of the participants or their training experience. The primary outcome measures were maximal oxygen uptake (VO_2_max), repeated sprint ability (RSA), change of direction speed, speed, explosive strength and body composition.

**Results:**

A total of 13 studies met the inclusion criteria, with a total of 230 participants. HIIT improved VO_2_max in five studies (ES from 0.19 to 1.08), while three studies showed improvement in their RSA (ES from 0.32 to 0.64). In addition, change of direction speed was improved in five studies (ES from 0.34 to 0.88), while speed improved in four studies (ES from 0.12 to 0.88). Explosive strength results varied (ES from 0.39 to 1.05), while in terms of body composition, the results were inconsistent through observed team sports.

**Conclusion:**

HIIT has significant effects on VO_2_max, RSA, change of direction speed, speed and explosive strength in female team sports, regardless of the competition level.

## Key Points

- High-intensity interval training (HIIT) is a time-effective approach for improving physical performance in female team sports.
- HIIT methods optimize both pre-season and in-season performance in different female team sports.
- In order to optimally develop sports performance, HIIT includes a variety of loads and training types, depending on the sport.

## Introduction

High-intensity interval training (HIIT) is considered to be an effective type of training for improving both metabolic and cardiovascular functions of athletes [1, 2]. At the same time, it is a time-efficient strategy for endurance development and short-term maximal performance, an important prerequisite for success in team sports [3]. In addition, HIIT simultaneously supports specific requirements of team sports that will be realized in the competition itself [4, 5]. It is well known that female team sports have intermittent character with often changes of low- and high-intensity periods [6–9]. Consequently, HIIT creates more high-intensity stimuli closer to female team sports requirements, such as soccer, basketball or handball compared to continuous training [10]. Despite HIIT being a very popular type of training in team sports, much more scientific attention is given to a systematic review summarizing the effects of HIIT on physical performance in non-athlete population compared to team sports athletes.

A previou﻿s﻿ systematic review [11] and meta-analysis confirmed that HIIT may improve aerobic and anaerobic performance of young athletes to a greater extent compared to alternative training programs and, at the same time, requires less time per training session. In addition, Manuel Clemente et al. [12] concluded that HIIT significantly improved VO_2_max, field-based aerobic performance and repeated sprint ability (RSA), while sprinting time remained unchanged in male soccer players. Finally, a meta-comparison of the effects of HIIT to those of small-sided games (SSG) and other training protocols indicates that HIIT and SSG have equally beneficial effects on endurance and soccer-specific performance of young male soccer players, but little influence on neuromuscular performance [3]. However, all aforementioned reviews and meta-analyses have dominantly included male team sports athletes. Accordingly, there are insufficient results for a conclusion about the effects of HIIT in female team sports athletes, because studies of female participants account for only 20% of all sport and exercises science research [13]. Additionally, it is unclear whether HIIT produces similar, the same or completely different effects among female team sports, depending on the intervals selected, the type of sport, the athlete’s motivation or the influence of the menstrual cycle for example. Some authors believe that female-specific physiology, such as the variations in female hormone concentrations throughout the menstrual cycle, may be a significant factor for maximizing performance [14, 15], contrary results have also been reported [16].

Female team sports have increased in popularity and urgently need the same treatment as male sports, but much more attention is still devoted to male athletes even in practice. In addition, applying results from studies on male to female athletes may be erroneous [14]. Schmitz et al. [17] have concluded that HIIT protocols may not be used interchangeably between males and females due to higher fatigue rates in females and anthropometric and physiological differences between the sexes, which may affect training performance in real-world settings. All previous HIIT systematic reviews in team sports were focused on male soccer players [3, 12] or young athletes [11], and there has been no review that systematically analyzes the effect of HIIT on physical performance in female team sports athletes. Therefore, the aim of this systematic review was to summarize the effects of HIIT on physical performance in female team sports athletes.

## Methods

### Literature Identification

Search and study analysis was done in accordance with PRISMA guidelines [18, 19]. The research included studies published prior to September 2nd, 2022. Computerized literature searches were conducted on the following databases: Google Scholar, PubMed, Web of Science, Cochrane Library, ProQuest and Science Direct.

The search was performed covering the areas of HIIT and female team sports using the following key terms and strings, either individually or combined: (“HIIT” OR “high-intensity interval training”) AND (“women” OR “female”) AND (“female team athletes” OR “handball” OR “basketball” OR “volleyball” OR “soccer” OR “futsal”) AND (“physical performance” OR “maximal oxygen uptake” OR “VO_2_max” OR “repeated sprint ability” OR “agility” OR “speed” OR “explosive power” OR “explosive strength” OR “body composition”). The literature search, quality assessment and data extraction were carried out, as well as the reference lists reviews from the downloaded studies. After the cross-review, the identified studies were rejected or taken for further analysis.

Two independent reviewers performed the literature search, identification, screening, quality assessment and data extraction. The reviewers initially screened titles, then abstracts and finally full texts to assess suitability of papers and all papers beyond the scope of this systematic review were excluded. In addition, the reference lists from the retrieved manuscripts were also examined for any other potentially eligible papers. Any disagreements between the reviewers were resolved by consensus or arbitration by the third reviewer. The corresponding author was directly contacted via email if a full text was not available.

### Inclusion Criteria

Type of study and participants: Longitudinal, randomized and matched controlled trials investigating HIIT interventions were included in the systematic review without any restrictions regarding publication date.

Included participants were female athletes engaged in team sports in an elite, sub-elite or college level of competition. Basic fitness level and experience were not set as inclusion criteria. Recreational female team sports studies, as well as studies with mixed sex without enough information for each sex separately, were excluded from further analysis.

*Type of intervention* Training programs had to last at least 2 weeks, there had to be at least one experimental group, and the intensity had to be at 80–100% maximal heart rate (HRmax). The number of trainings per week was not considered as an inclusion criterion. Studies with the combination of HIIT and any other training type which can impact overall outcomes were excluded.

*Type of outcome measures* The primary outcome measures for the systematic review were VO_2_max, RSA, change of direction speed, speed, explosive strength and body composition.

### Risk of Bias Assessment

The risk of bias was assessed according to the PRISMA recommendation, i.e., the PEDro scale [20] was used to determine the quality of the studies and the potential risk of bias. The score scale shows the validity result of each study individually in the range from 0 (high risk of bias) to 11 (low risk of bias), and the points are awarded only when the criteria are fully met. Two independent reviewers assessed quality and risk of bias using checklists. Agreement between the reviewers was assessed using *k* statistical data to screen the entire text, assess relativity and risk of bias. In cases of disagreement about the risk of bias, the verification of the data was performed by the third reviewer who made the final decision. The *k* rate of agreement between reviewers was *k* = 0.93.

### Data Extraction

The data were extracted by two authors independently, while the third author cross-reviewed the data for accuracy and completeness. Any disagreements were resolved by a consensus or by the third and fourth reviewer. Then, the data were extracted and transferred to an Excel spreadsheet. Cochrane Consumer and Communication Review Group’s standardized data extraction protocol [21] was used to extract study characteristics including the author(s), title and year of publication; participant information such as sample size, age and sex, description of the training intervention, including intensity, duration and frequency, and study outcomes, including the following physical performance variables: VO_2_max, repeated sprint ability, change of direction speed, speed, explosive strength and body composition. The reviewers were not blinded to authors, institutions or manuscript journals.

## Results

### Study Selection and Characteristics

**Fig. 1:**
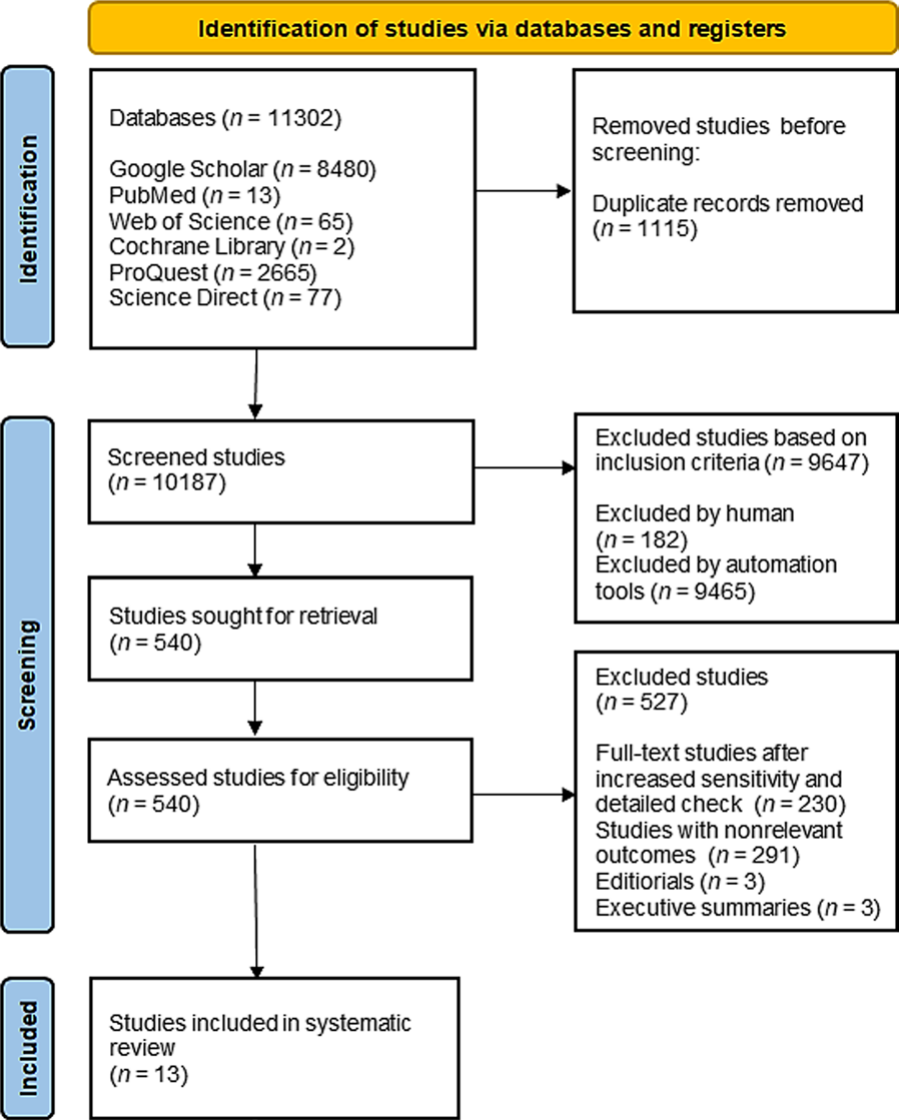
Included studies in the systematic review based on pre-defined criteria (PRISMA flow chart)

The electronic databases search and scanning of reference lists of articles revealed a total of 11,302 relevant studies. Firstly, 1115 studies were excluded as duplicates. Further, a total of 10,187 studies were screened, of which 9647 studies were excluded based on the inclusion criteria. Then, 540 full-text studies were assessed for eligibility and taken into consideration, of which 527 were additionally excluded, based on more in-depth checking, identification of non-relevant outcomes, or because they were editorials or executive summaries. Lastly, a total of 13 full-text studies were included in the systematic review (Fig. 1).

Each study was read and coded for descriptive variables: age, sample size, sport, duration, type of control group (no exercise, regular training), mode of exercise and intensity of control group exercise, manner and intensity exercise of experimental group, work—rest ratio, rest ratio and length of intervention, as well as the outcome of the study.

The included studies 230 female participants in total. The studies included participants from different team sports: basketball [9, 22, 23], handball [24, 25] soccer [8, 26, 27], futsal [28, 29] and volleyball [30]. The remaining two studies included participants from ice hockey [31] and field hockey [32]. The duration of the programs varied from 2 to 10 weeks. In addition, the highest number of participants in one study 37 [8], while the lowest was only 11 [30] (Table 1). Table 2 describes the studies with pre-defined research outcome measures.

**Table 1:** Studies included in the qualitative analysis

Study	Participants		Duration (weeks)	Program (type, intensity frequency, training duration)	Measured outcomes	Results				
	Age (years) and sport	Number and groups								
Aschendorf et al. [9]	15.1 ± 1.1 Basketball	*N*-24 *E*-11 *C*-13	5	*E*-specific basketball HIIT; 90–95% HRmax; 2 × a week; 25 min *C*-maintained a normal training	BF FFM CMJ CMJa SJ YYIR1 COD Shuttle run 20 m	*E*		*C*		
						COD180 ↑ CMJ ↔ CMJa ↔ SJ ↔ YYIR1 ↑ BF ↔ FFM ↔		COD180 ↓* CMJ ↔ CMJa ↔ SJ ↔ YYIR1 ↔ BF ↔ FFM ↔		
Sanchez-Sanchez et al. [22]	17.2 ± 1.1 Basketball	*N*-12 HITCOD1-6 HITCOD3-6	6	HIITCOD1-90% Vift; 2 × a week on 6 regular trainings; 2 × 6 min HIITCOD3-90% Vift; 2 × a week on 6 regular trainings; 2 × 6 min	V-cut RSA MAT Vift	HIITCOD1		HIITCOD3		
						V-cut ↑ RSA ↑ Vift ↑		MAT ↔ V-cut ↑* RSA ↑* Vift ↑*		
Zeng et al. [23]	19.9 ± 1.1 Basketball	*N*-19 SSG-9 HIIT-10	4	3 × (6–9 min of 15′–15′ at 90–95% of Vift); 3 × a week	20 m CMJ MAT RSA Vift	SSG		HIIT		
						20 m ↑ CMJ ↑ MAT ↑* RSA ↑* Vift ↑*		20 m ↑ CMJ ↑ MAT ↑* RSA ↑* Vift ↑*		
Alonso-Fernandez et al. [24]	15.2 ± 0.6 Handball	*N*-14 *E*-7 *C*-7	8	E-HIIT C-regular warm-up; HR = 85% + ; 2 × a week; 1–2 week: 4′ 3–4 week: 4′2′4 5–6 week: 4′2′4′2′4′ 7–8 week: 4′2′4′2′4′2′4′	BMI BF CMJ VO_2_max	E		C		
						VO_2_max ↑* CMJ ↔ BMI ↔ BF ↑*		CMJ ↔ BMI ↔		
Jurišić et al. [25]	16.20 ± 1.28 Handball	*N*-24 SSG-12 HIIT-12	8	2 × (6–8 min of 15′–15′ at 90–95% of MAS with 3 min. recovery between series); 2 × a week	CMJ SJ 20 m 30 m YYIR1	SSG		HIIT		
						CMJ ↑* SJ↑* 20 m↑* 30 m ↔ YYIR1 ↑*		CMJ ↑* SJ ↑* 20 m ↑* 30 m ↑* YYIR1 ↑*		
Rowan et al. [27]	19.4 ± 0.87 Soccer	*N*-15 STR-8 COD-7	2	STR(1–3)–7 × 30 m; 3 series STR (4–6) 4 series COD(1–3)–7 × 20 m; 3 series COD (4–6) 4 series; 2 weeks; 3 × a week	VO_2_max YYIR1	STR		COD		
						VO_2_max↑* YYIR1 ↑*		VO_2_max↑* YYIR1 ↑*		
Wright et al. [8]	13.4 ± 1.5 Soccer	*N*-37 E1(pre-PHV)-6 E2(at PHV)-7 E3(after PHV)-24	8	All groups worked 2 blocks for 4 weeks #1 block-longer HIIT #2 block-SIT 1 block-1 week-1 training 2–4 week-2 trainings #2 block-5 week-2 trainings 6–7 week-2 trainings 8 week-1 training + individual training for all (1 × 70 min)	20 m test YYIR1	E1	E2			E3
						20 m↓* YYIR1↑	20↑m YYIR1↑			20 m↓* YYIR1↑*
Arazi et al. [26]	23.4 ± 1.3 Soccer	*N*-16 *E*-8 *C*-8	6	(E) HR HIIT-90% from maximal speed (C) HIIT-90% from maximal speed (3 × a week + regular trainings)	VO_2_max Vift	(E) HR HIIT			(C) HIIT	
						VO_2_max↑* Vift ↑*			VO_2_max↑* Vift ↑*	
Kinnunen et al. [31]	22 ± 3 Ice hockey	*N*-14	2	2 × a week HIIT 6 × 30c sprint with an ascent of 9.5% with a break of 4 min between each series	CMJ SJ 11 m skiing 34 m skiing	11 m skiing ↔ 34 m skiing ↔ CMJ ↔ SJ ↔				
Funch et al. [32]	19.29 ± 0.91 Field hockey	*N*-14 HIIT(run)-8 HIIT(Tab)-6	4	12 trainings HIIT(run) 30 min run HIIT(Tab) 4 min (75–85%)	VO_2_max	HIIT(run)			HIIT(Tab)	
						VO_2_max↑*			VO_2_max↑*	
Teixeira et al. [29]	18.71 ± 1.94 Futsal	*N*-14 SRIT1-7 SRIT3-7	5	SRIT1 (89%)-4 × 4 min; 3 min rest (7.5 s run–7.5 s rest) 16 shuttles; SRIT3 (86%)-4 × 4 min; 3 min rest (15 s run-15 s rest); 8 shuttles	40 m Shuttle CMJ SJ VO_2_max	SRIT1			SRIT3	
						40 m Shuttle ↑ VO_2_max↑ CMJ ↑* SJ ↑*			40 m Shuttle ↔ VO_2_max↑ CMJ ↑ SJ ↑	
Teixeira et al. [28]	19.2 ± 2 Futsal	*N*-16 SRIT1(7.5 × 7.5)-7 SRIT3(15 × 15)-9	5	SRIT1 (89%)-4 × 4 min; 3 min rest (7.5 s run-7.5 s rest); 16 shuttles; SRIT3 (86%)-4 × 4 min; 3 min rest (15 s run-15 s rest); 8 shuttles	RSA VO_2_max	SRIT1(7.5 × 7.5)			SRIT3(15 × 15)	
						RSA↑* VO_2_max↑			RSA↑* VO_2_max↑	
Afyon et al. [30]	15.27 ± 1.10 Volleyball	*N*-11 *E*-6 *C*-5	6	E-Tab; 2 × a week; 8 min C- maintained a normal training	20 m test 20 m Shuttle CMJ	20 m ↑* 20 m shuttle ↑* CMJ ↑				

*N*—total number of participants; *E*—experimental group; *C*—control group; HIITCOD1/3—high-intensity interval training with 1 or 3 change of directions; Vift—capacity at the last completed interval; V—cut-25 m maximum running with 4 changes of direction (4 cones); MAT—modified agility test; SRIT1/3—shuttle run interval training with 1 or 3 changes; Tab—Tabata training; PHV—pick height velocity; RSA—repeated sprint ability; STR—sprint training; SIT—sprint interval training; COD—change of direction; SSG—small-sided games; CMJ—countermovement jump; SJ—squat jump; CMJa—countermovement jump with arm swing; VO_2_max—maximal oxygen consumption; YYIR1—YoYo Intermittent Recovery Test Level 1; Vift—final 30–15 intermittent test velocity; MAS—maximal aerobic strength; BMI—body mass index; FFM—fat-free mass; BF—body fat; HR—heart rate; HRmax—maximum heart rate; COD180—change of direction for 180°; ↑—statistical significance (*p* < 0.05); ↑*—statistical significance (*p* < 0.01); ↓—statistical decrease (*p* < 0.05); ↓*—statistical decrease (*p* < 0.01); ↔—maintaining result

**Table 2:** Research output

Study	*N*	Sport	VO_2_max	RSA	*A*	*S*	ES	BC	∑
Aschendorf et al. [9]	24	Basketball	−	−	+	−	+	+	3/6
Sanchez-Sanchez et al. [22]	12	Basketball	−	+	+	−	−	−	2/6
Zeng et al. [23]	19	Basketball	−	+	+	+	+	−	4/6
Alonso-Fernandez et al. [24]	14	Handball	+	+	−	−	+	+	4/6
Jurišić et al. [25]	24	Handball	−	−	−	+	+	−	2/6
Rowan et al. [27]	15	Soccer	+	−	−	−	−	−	1/6
Wright et al. [8]	37	Soccer	−	+	+	+	−	−	3/6
Arazi et al. [26]	16	Soccer	+	−	−	−	−	−	1/6
Kinnunen et al. [31]	14	Ice hockey	−	−	−	+	+	−	2/6
Funch et al. [32]	14	Field hockey	+	−	−	−	−	−	1/6
Teixeira et al. [29]	14	Futsal	−	−	−	+	+	−	2/6
Teixeira et al. [28]	16	Futsal	+	+	−	−	−	−	2/6
Afyon et al. [30]	11	Volleyball	−	−	−	+	+	−	2/6
**∑**	230		5	5	4	6	7	2	

*N*—total number of participants; RSA—repeated sprint ability; *A*—agility; *S*—speed; ES—explosive strength; BC—body composition; ∑—total score

### Study Quality

From the total number of the studies that entered the qualitative analysis, based on the points earned by each study on the PEDro scale, the final results of the study quality assessment were defined. Only one study was of poor quality, three studies were of fair quality, and the other 9 studies were of good quality. The final results of the study quality assessment are presented in Table 3.

**Table 3:** PEDro scale results

Study	1	2	3	4	5	6	7	8	9	10	11	∑
Aschendorf et al. [9]	Y	N	Y	Y	N	N	N	Y	Y	Y	Y	6
Sanchez-Sanchez et al. [22]	Y	Y	Y	Y	N	N	N	Y	Y	Y	Y	7
Zeng et al. [23]	Y	Y	Y	Y	N	N	N	Y	Y	Y	Y	7
Alonso-Fernandez et al. [24]	Y	Y	Y	Y	Y	N	N	Y	Y	Y	Y	8
Jurišić et al. [25]	Y	N	N	Y	N	N	N	Y	Y	Y	Y	5
Rowan et al. [27]	Y	Y	Y	Y	N	N	N	Y	Y	Y	Y	7
Wright et al. [8]	Y	N	Y	N	N	N	N	N	Y	N	Y	3
Arazi et al. [26]	Y	Y	Y	Y	N	N	Y	Y	Y	Y	Y	8
Kinnunen et al. [31]	Y	Y	N	N	N	N	N	Y	Y	N	Y	4
Funch et al. [32]	Y	Y	Y	Y	Y	N	N	Y	Y	Y	Y	8
Teixeira et al. [29]	Y	Y	N	Y	N	N	N	Y	Y	Y	Y	6
Teixeira et al. [28]	Y	Y	N	Y	N	N	N	Y	Y	Y	Y	6
Afyon et al. [30]	Y	N	N	N	N	N	N	Y	Y	Y	Y	4

1—eligibility criteria; 2—random allocation; 3—concealed allocation; 4—baseline comparability; 5—blind subject; 6—blind clinician; 7—blind assessor; 8—adequate follow-up; 9—intention-to-treat analysis; 10—between-group analysis; 11—point estimates and variability; Y—criterion is satisfied; *N*—criterion is not satisfied; ∑—total awarded points

### Effects of HIIT on VO_2_max

In all five studies that analyzed the impact of HIIT on VO_2_max, the results showed that the best effects (17.3%, ES = 0.71) were found after a six-week speed-based HIIT program in soccer players [26], while the least effects were found following a five-week training program in futsal players (HIIT 7.5 × 7.5 s − 1.77%, ES = 0.19); HIIT 15 × 15 s − 2.32% (ES = 0.22) [28]. Regardless of the variety of the tests, there was also a positive effect in handball players of 6.2% (ES = 1.01) [24], soccer players 4.7% [27] and field hockey players 6.1% (ES = 0.78) for running-based HIIT and 6.2% (ES = 1.08) for Tabata [32]. Non-significant changes in VO_2_max (1.26%) were observed in the control group [24].

### Effects of HIIT on RSA

One study [28] with two different protocols showed that the group with a longer distance (15 × 15) showed improvement of 3.6% (ES = 0.60), while the group with a shorter distance (7.5 × 7.5) improved the test time (8 × 40 m) by 2.6% (ES = 0.32). Basketball players [22] improved their time of repeated sprint ability (6 × 20 + 20 m) by 4.5% in the group with 3 turns, while in Zeng et al. [23], basketball players also improved pre-post time for RSA_mean_ (1.9%, ES = 1.0) and RSA_best_ (2.1%, ES = 1.1). Soccer players [8] had large improvements (6.5%) in the before-PHV (peak height velocity) group following a mixed methods HIIT, while the at-PHV group showed moderate improvements (1.7%) in 3 × 20 m RSA test.

### Effects of HIIT on Change of Direction Speed

The obtained results showed that HIIT significantly improved COD time in basketball players [9, 22, 23], where the change in magnitude was in the range of 1.6 to 6.2%. In the first case, basketball players completed the shuttle sprint test (ES = 0.34) (20 m COD), in the second the V-cut test (2.4–4.8%), and in the third case the Modified Agility Test (ES = 1.55). In addition, a HIIT program in soccer players [8] achieved 2.7% improvement on the T test. The result in the control groups was different. In one study [9], a decrease in the results achieved in the COD test of 1.21% was recorded, while the control group SSG (small-sided games) showed an improvement of 7.2% on the MAT test [23].

### Effects of HIIT on Speed

The obtained results showed that the HIIT program led to a significant improvement in 20-m sprint time in volleyball players by 4% (ES = 0.88) [30]. Small improvements (1.4%) were found in young soccer players [8], while ice hockey players [31] reduced their skating time over 11 m by 1.5% (ES = 0.27) and over 34 m by 0.6% (ES = 0.12) after speed-based HIIT training. The futsal players in the group with one change of direction [29] after the HIIT program improved the time on the speed test by 1.1% (ES = 0.33), while the group of participants with 3 turns achieved slightly better results (2.9%, ES = 0.58). Furthermore, in Jurišić et al. [25], handball players had a 1.94% improvement in 20-m sprint (ES = 0.54). In addition, in Zeng et al. [23], basketball players slightly improved their 20-m sprint by 1.3% (ES = 0.20).

### Effects of HIIT on Explosive Strength

Most of the studies showed no statistically significant changes for the CMJ test—small to moderate changes (0.7–9%). In two studies, the results show that HIIT had positive effects on CMJ in volleyball players, of 7.8% (ES = 0.61) [30] and futsal players (group with 3 turns 8.4% (ES = 0.66) and group with 1 turn 7% (ES = 1.05)) [29]. In Zeng et al. [23], basketball players also slightly improved CMJ (5.4%, ES = 0.47), with a small effect size (*d* = 0.5), while in Jurišić et al. [25], handball players had a slight increase in sprint time (2.63%; ES = 0.47) [*p* = 0.01]. Regarding the SJ test, HIIT showed a positive effect (4.8%, ES = 0.39) in ice hockey players [31] and futsal players (group with 1 turn 7.8% (ES = 0.89) and group with 3 turns 9.2%, ES = 0.69) [29].

### Effects of HIIT on Body Composition

The body fat of basketball players remained unchanged [9], in contrast to handball players [24] who had a positive effect on the same variable of 3.4% (ES = 1.49), while the BMI of these handball players remained unchanged (− 0.5%). Non-significant changes in control groups (BMI: − 1.8%; body fat: − 1.25% [24]; body fat: 0%; body weight: − 0.015% [9]) were also observed.

## Discussion

This systematic review has revealed that HIIT can improve physical performance in female team sport athletes. Specifically, VO_2_max showed the best improvements in team sports athletes regardless of the sports discipline [24, 26–28, 32]. Additionally, improvements were also seen in RSA, change of direction speed, speed and explosive strength. In terms of body composition, results were inconsistent through observed team sports. Compared to the control groups, the HIIT groups had more effective results in all cases. In two cases [22, 28], the groups with a higher number of CODs achieved greater improvements compared to the groups with fewer CODs, while in one case the results of the groups were similar [29].

It is widely accepted that HIIT offers significant benefits for improving aerobic endurance [33]. It is also a good strategy to improve the aerobic performance of players over a short period of time [34–36]. This was confirmed by two studies in futsal and soccer players that lasted 5 weeks, with two training sessions per week. Futsal players [28] improved their VO_2_max using two interval periods, 7.5 and 15 s, while soccer players [27] used 5 × 30-s maximum effort sprints with 4.5- and 3.5-min active recovery. In addition, a longer duration (8 weeks) HIIT program, which consisted of the Tabata protocol in handball players [24], showed improvement in VO_2_max of 6.1%. The Tabata protocol was used also in field hockey players [32] with similar improvements. Although different training mechanisms and different types of HIIT training were used, similar responses from athletes were obtained regarding aerobic performance. In addition, short intervals of HIIT allow the volume and intensity to be manipulated, while HIIT with long intervals stimulates the work of the anaerobic system and neuromuscular load [10]. Anaerobic glycolytic energy is a substantial component of the short intervals. Furthermore, field-based HIIT formats with short intervals are linked to lower initial blood lactate accumulation rates than those with long intervals [10]. Despite the fact that a recent review had difficulties drawing stronger conclusions regarding the importance of exercise intensity for cardiovascular adaptations [37], the current findings may be considered relevant in these team sports. Additionally, greater improvements can be obtained in a shorter time compared to traditional endurance training [2]. This finding is also confirmed in the current review, where very short (≤ 15 s) [22, 23, 25, 28, 29] and short intervals (≤ 60 s) [8, 9, 24, 27, 30, 32] presented greater improvement. Likewise, in the studies where HIIT frequency within the interventions was over 90%, improvements were present as well [9, 22, 25, 26, 38].

Different HIIT protocols showed improvement in RSA performance. HIIT with one and three turns produced similar effects for RSA (8 × 40 m) in futsal players [28], as well as in basketball players [22] (6 × 20 m), with a similar change of direction training method used. These findings confirm that well-prepared athletes from different team sports show a similar and positive result for RSA which was probably due to the related ability to develop maximum speed in these team sports [39]. One study [40] emphasizes the importance of the magnitude of accelerations and time spent accelerating per running bout, so it seems logical to assume that these players had more powerful accelerations and spent more time accelerating per running bout. These more powerful accelerations also elicit greater neural activation of the working muscles [41, 42].

The ability to change direction during high-intensity running has been recognized as an important factor for successful participation in team sports [10, 43, 44]. The directional changes involved in HIIT training increase the specificity of the training process, which mimics the situations in the game and increases cardiorespiratory, neuromuscular and perceptual responses when performing these movements [40, 45]. Therefore, the inclusion of a change of direction in HIIT becomes an inevitable part of everyday training [46, 47]. The results of the present review showed that female players [9, 22] significantly improved their performance time in change of direction speed tests, with also minor improvements occurring on the tests performed with the ball [9]. Also, the more successful groups in the studies had an experimental program that had 3 changes of directions, which requires the participants to accelerate and decelerate on more occasions. It is important not to omit additional upper-body muscles and eccentric muscular contractions [44], which provide a greater stimulus to improve neuromuscular and metabolic factors [10]. Soccer players [8] showed only minor improvements in change of direction speed, mainly because these participants were high level, and it was expected that the improvements would be relatively small. Nevertheless, these minor improvements may suggest that 8-week mixed-method HIIT training interventions elicited neural adaptations. This factor could be important for physical performance and injury prevention during in-season, given the role of neuromuscular control [48]. Adjusted distances, number, intensity and the nature of the runs, with also a duration of recovery should be taken into consideration, especially during in-season [10]. The current results show that the best effects of HIIT training were with three turns [22], while responses to other methods were different. Given the demands of the team sport regarding the ability to change direction during high-intensity running, the main focus should be on endurance and metabolic conditioning in female players.

High-intensity running performance is important, given that it is proposed to occur in close proximity to key moments in competition [49]. However, only the Tabata training program in female volleyball players [30] significantly improved speed in the 20-m sprint test. Because of the nature of this training program, which consisted of 20 s with maximum load and 10 s of active rest, the presented short interval training showed adaptations, which lead to improvements in speed. Furthermore, neuromuscular adaptation is also proposed to be one of the key adaptations leading to improved performance in power activities, such as sprinting [50]. In contrast to this result, minor improvements have occurred in soccer players [8] and futsal players [29]. The variety of results indicates that maturation can greatly affect the results, because when assessing physical fitness, growth and maturation are considered the main confusing factors [51], since these players ranged from 13.4 to 18.7 years old﻿. In addition, futsal players [29] had an improvement in speed after HIIT in the preparatory phase, which should be taken into account. A large number of accelerations are an adequate and effective training model that leads to strengthening and increasing the capacity of leg muscle strength, fatigue index and endurance of the players, causing large adaptive responses in the properties of muscle fibers [52]. Ice hockey players [31] showed improvements during the program that lasted only 2 and a half weeks. Fast adaptations, regarding muscle fiber recruitment, frequency and motor unit synchronization, could provide some explanation for the rapid improvements observed [53], but the mechanism for ﻿the results obtained should be further investigated in future studies.

It is generally accepted that explosive strength is an important factor in both individual and team sports. To improve CMJ, similar training programs were used in handball [24] and basketball players [22], which consisted of 20 s of work and 10 s of rest [54]. Although similarities in HIIT protocols were present, there were some contradictory results [7, 46]. HIIT induced significant improvements in CMJ among futsal players [29] in both monitored groups, with one and three turns. The results obtained by Iacono et al. [55] also showed significant improvements in handball players, while some other studies [56, 57] found opposite results. Since the above mentioned futsal players [29] also had improved speed, a possible explanation for these results might be the fact that with the increased demands of sprinting ability, explosive strength also increases [58]. Ice hockey players [31] maintained their CMJ results for only two and a half weeks after the completion of the training program. This is most likely due to the fact that the subjects of the study were highly skilled skaters in whom CMJ height did not change, suggesting that improvements affected mainly the concentric force production according to the authors. Therefore, despite neural and functional development, a longer training period should be used to improve vertical jump performance [59], which could be considered in future studies. Similarly, basketball players [9] also maintained their CMJ and CMJa (countermovement jump with arm swing) results [60, 61]. However, it should be mentioned that HIIT program was conducted during the in-season. Moreover, the design of the exercise was with 180-degree turns, where participants might have turned on their preferred side, thus promoting unilateral adaptations which might not be manifested in bilateral activities such as vertical jumping. Improvements in SJ were observed in futsal [29] and ice hockey players [31]. Several factors may contribute to changes in muscle performance during jumping performances, such as an increase in muscle capacity to develop higher tension, to add more contractile elements or to store and reuse elastic energy [62]. From a practical point of view, the choice of a HIIT training depends to a large extent on the time point during the season and the coach’s overall strategy [3].

Increasing lean body mass is one of the most important mediators for improving strength and power, relevant to sports performance [63]. HIIT reduced the body fat of handball players [24] after 8 weeks, and this result is in accordance with improvements in football players [64], where only 3 weeks of HIIT showed similar results. However, basketball players showed the opposite results [9], with unchanged body composition after 5 weeks. Considering the phase of the training process and the limited time for the changes in body composition, it is often difficult to make a difference, especially in female athletes. Such differences can be caused also by differences in sports, individual differences of athletes, type of training, and the metabolic or physiological requirements of different sports [65].

Improving fitness is a complex process, which requires a lot of planning [60]. High-intensity actions such as jumping, accelerating, slowing down, various sprints with changes of direction [66] and the athlete’s ability to repeat these actions identically in the competition are the key to achieving success in many team sports such as soccer, basketball, handball or volleyball [67]. To this end, athletes need to optimally develop sports performance, aiming for adequate levels [66, 68], and in order to achieve this successfully, it is necessary to include different loads and different types of training, depending on the sport.

Due to the differences in HIIT protocols, including intensity, duration, frequency, rest durations and different control conditions, as well as sports diversity and level of competition, the authors cannot with certainty determine an effective HIIT protocol, which could be the main limitation of this review. Another limitation can be attributed to the fact that these results should be interpreted with caution, since HIIT protocols were implemented during both in- and out of season. Furthermore, we have not factored in the possible impact of the menstrual cycle on physical performance results. Due to the small number of included studies, this study demonstrates that the growing and relevant population of female team sports athletes is, currently, an unexplored group in exercise science, which should have implications for future research.

## Conclusion

It can be concluded that HIIT programs, regardless of the type, induce improvements in VO_2_max, RSA, change of direction speed, speed, explosive strength of the lower limbs and body composition in female athletes who are engaged in team sports. Regardless of the level of training or competitive experience, HIIT offers benefits in both the preparation period, when physical performance is raised to a higher level, and in the competitive period, where it can be maintained. It is also highly important that coaches use HIIT methods when preparing their teams and adjust the HIIT type according to the time of the season when it is used.

## Data Availability

Not applicable.
